# Functional mechanisms, current applications, and future perspectives of dairy products in oral health

**DOI:** 10.3389/fpubh.2026.1898007

**Published:** 2026-08-31

**Authors:** Miao Wang, Qian Wang, Bin Chen, Jianguo Liu

**Affiliations:** 1 Key Laboratory of Microbial Resources and Drug Development of Guizhou Education Department, School of Stomatology, Zunyi Medical University, Zunyi, China; 2 Key Laboratory of Oral Disease Research of Guizhou Education Department, School of Stomatology, Zunyi Medical University, Zunyi, China

**Keywords:** dairy products, dental caries, oral health, periodontitis, probiotics, public health nutrition, CPP-ACP

## Abstract

**Background:**

Oral diseases affect over 3.5 billion people globally, with dental caries and periodontitis being the most common. Previous studies suggest that dairy products may protect against these conditions through enamel remineralization and antimicrobial effects, but a comprehensive synthesis integrating component profiles, molecular mechanisms, and translational applications has been lacking. This systematic review synthesizes evidence on dairy products and their bioactive components in oral health, clarifies functional mechanisms, current applications, and future directions.

**Methods:**

PubMed, Cochrane Library, and China National Knowledge Infrastructure (CNKI) were searched from database inception to April 2026. Studies linking dairy products to oral health were included. A narrative synthesis covered dairy product categories, bioactive components (proteins and peptides, minerals, probiotics), mechanisms (microecological regulation, caries prevention, periodontal management, antifungal effects, mucosal protection), current applications, and future challenges.

**Results:**

Processing characteristics and compositional differences of liquid milk, fermented dairy products, and cheese determine their distinct roles in oral care. Dairy-derived proteins, minerals, and probiotics act synergistically through multiple mechanisms: maintenance of oral microbial homeostasis; enamel remineralization, inhibition of cariogenic bacterial adhesion and acid production (caries prevention); attenuation of inflammatory responses (periodontal support); inhibition of Candida albicans (opportunistic infection control); and protection of mucosal barrier integrity. A three-tiered application model–natural dietary intervention, oral care product development, and clinical adjunctive therapy–has emerged. This review adds an integrated “components–mechanisms–effects” framework demonstrating how casein phosphopeptides, lactoferrin, probiotics, calcium, and phosphorus synergistically maintain oral microbiota homeostasis, promote remineralization, reduce inflammation, inhibit opportunistic infections, and protect the mucosal barrier.

**Conclusion:**

Dairy products can preserve oral health via multiple pathways and have clear public health value. Future research should focus on probiotic strain specificity, synergistic mechanisms of active ingredients, and long-term clinical effects to promote an integrated “nutrition + oral care” health management strategy. This review provides a theoretical reference for precise application, product innovation, and interdisciplinary integration of dairy products in oral health, with positive implications for improving global oral health status.

## 1 Introduction

Oral diseases are among the most common chronic non-communicable diseases worldwide. According to the World Health Organization's Global Oral Health Status Report (1), more than 3.5 billion people are affected by oral diseases, including approximately 2.5 billion with dental caries and 514 million children with untreated caries in primary teeth. The annual global cost of treating oral diseases exceeds US$ 442 billion, placing a heavy burden on public health systems. Periodontitis, another highly prevalent oral disease, not only damages the supporting tissues of the teeth but is also closely linked to various systemic conditions, including diabetes mellitus, cardiovascular disease, chronic kidney disease, and obesity (2), further highlighting the importance of maintaining good oral health.

Currently, the prevention and control of oral diseases rely mainly on professional interventions such as pit-and-fissure sealants and fluoride applications. The joint guideline of the American Dental Association and the American Academy of Pediatric Dentistry recommends pit-and-fissure sealants as a first-line measure for caries prevention in permanent teeth of children and adolescents. Nevertheless, their uptake remains suboptimal: the coverage of sealants among US school-aged children is only 42%, and less than 30% among those at high caries risk. Moreover, such interventions have limitations including insufficient protection of smooth surfaces, dependence on professional administration, and poor accessibility to dental care (3, 4). The US national oral health objectives (Healthy People 2020) explicitly set targets such as “increase the proportion of children who receive dental sealants” and “reduce the proportion of people with dental caries,” underscoring the urgent need for low-cost, easy-to-implement approaches to supplement traditional prevention strategies (5).

Diet is a key environmental factor influencing oral health, and adequate nutrient intake plays an essential role in preventing oral diseases and maintaining oral function (6). Dairy products are an important component of the human diet. They not only provide high-quality protein, calcium, phosphorus and other basic nutrients but also contain a variety of bioactive components such as lactoferrin, casein phosphopeptide (CPP), and probiotics. Numerous studies have confirmed their multi-dimensional protective effects on oral health, including enamel remineralization, inhibition of cariogenic bacteria, modulation of oral microbiota, and reduction of gingival inflammation (7–11). Compared with traditional professional interventions, dairy products are natural, convenient, accessible to all population groups, and easy to scale up, thus effectively complementing the limitations of existing measures.

Based on the above background, this systematic review aims to: (1) synthesize the mechanistic evidence explaining how dairy-derived bioactive components exert their effects on oral tissues; (2) summarize clinical and epidemiological evidence on the association between dairy consumption and oral health outcomes; and (3) translate these findings into practical recommendations for clinical practice and public health policy.

## 2 Methods

### 2.1 Study design

This systematic review was reported in accordance with the PRISMA 2020 statement (12). This systematic review was not prospectively registered in PROSPERO because data extraction had already been completed before registration was considered, making it impossible to meet PROSPERO's timeline requirements. No review protocol was prepared before conducting the review. The PRISMA 2020 checklist (27 items) is available in Supplementary Table S4.

### 2.2 Search strategy

A systematic search was performed in PubMed, Cochrane Library, and China National Knowledge Infrastructure (CNKI) from database inception to 15 April 2026. The English search terms were: (“dairy products” OR “milk” OR “yogurt” OR “cheese” OR “whey protein” OR “lactoferrin” OR “casein phosphopeptide” OR “probiotics”) AND (“oral health” OR “dental caries” OR “periodontitis” OR “candidiasis” OR “oral microbiota” OR “enamel remineralization”). The Chinese search terms were: (“乳制品” OR “牛奶” OR “酸奶” OR “奶酪” OR “乳清蛋白” OR “乳铁蛋 白” OR “酪蛋白磷酸肽” OR “益生菌”) AND (“口腔健康” OR “龋齿” OR “牙周炎” OR “口腔念珠菌病” OR “口腔微生态” OR “牙釉质再矿化”). In addition, the reference lists of included studies and relevant reviews were manually screened to identify additional eligible studies. Complete search strategies (including MeSH terms and field limits) for each database are provided in Supplementary Table S1 to ensure reproducibility.

### 2.3 Eligibility criteria

#### 2.3.1 Inclusion criteria

(1) study type: randomized controlled trials, non-randomized controlled trials, cohort studies, case-control studies, cross-sectional studies, systematic reviews/meta-analyses, *in vitro* studies, and animal experiments; (2) study population: any age, sex, or ethnicity; (3) intervention: dairy products (liquid milk, fermented dairy products, cheese, whey protein, milk-derived bioactive components, etc.); (4) outcome measures: caries incidence/prevalence, enamel remineralization, Streptococcus mutans counts, plaque index, gingival index, periodontal pocket depth, oral Candida colonization, oral mucosal healing, etc.; (5) language: English or Chinese. Exclusion criteria: (1) non-original research or non-systematic reviews (e.g., editorials, commentaries, case reports); (2) full text unavailable; (3) duplicate publications.

#### 2.3.2 Exclusion criteria

(1) non-original research or non-systematic reviews (e.g., editorials, commentaries, case reports); (2) full text unavailable; (3) duplicate publications.

**The research question was defined using the PICOS framework as follows:**

**P (Population):** human participants of any age, plus relevant *in vitro*/animal models for mechanistic questions.

**I (Intervention):** dairy products or their bioactive components (liquid milk, fermented dairy products, cheese, whey protein, CPP-ACP, lactoferrin, probiotics).

**C (Comparator)**: placebo, no intervention, standard care, or alternative products.

**O (Outcomes):** oral health outcomes, including mechanistic surrogates (e.g., bacterial counts, biofilm biomass) and clinical endpoints (e.g., caries incidence, periodontal parameters).

**S (Study designs):** all study designs providing relevant evidence for the specific sub-question, including RCTs, cohort studies, case-control studies, cross-sectional studies, *in situ* studies, *in vitro* studies, animal experiments, and systematic reviews.

**Rationale for including multiple study designs:** This review included multiple study designs to construct a complete “components–mechanisms–effects–applications” evidence chain. Mechanistic and preclinical studies (*in vitro*, animal, *in situ*) provide biological plausibility for the observed clinical effects, while clinical trials and observational studies provide direct evidence of effectiveness in human populations. Systematic reviews/meta-analyses served as sources for additional primary studies and as contextual references. Clinical evidence (RCTs and cohort studies) was prioritized for conclusions on clinical effectiveness, while mechanistic evidence was used only to support biological plausibility and was not weighted equally with clinical outcomes. This distinction is explicitly stated in the Methods section and reinforced in the Discussion.

### 2.4 Study selection and data extraction

Two investigators independently screened titles, abstracts, and full texts according to the eligibility criteria. Inter-rater agreement was assessed using Cohen's kappa statistic (κ = 0.89). Disagreements were resolved by discussion or consultation with a third investigator. The extracted data included: first author, publication year, country, study design, sample size, age range, intervention/exposure, comparator, key oral health outcomes, and results.

### 2.5 Quality assessment

Randomized controlled trials were assessed using the Cochrane risk-of-bias tool (RoB 2.0); cohort and case-control studies using the Newcastle-Ottawa Scale (NOS); cross-sectional studies using the National Institutes of Health (NIH) quality assessment tool; systematic reviews/meta-analyses using AMSTAR 2; and animal experiments using the SYRCLE risk-of-bias tool. Two investigators independently performed the assessments, and disagreements were resolved by discussion.

For *in situ* and non-randomized clinical studies that could not be evaluated with standard tools, we used an adapted checklist focusing on four domains: (1) random sequence generation (if applicable); (2) blinding of outcome assessment; (3) sample size adequacy; and (4) conflicts of interest. Two investigators independently assigned a judgment of “low,” “unclear,” or “high” to each domain. The overall judgment was then derived as follows: **low risk** = all domains rated low; **moderate risk** = one domain rated unclear or two domains rated low but with minor concerns; **high risk** = two or more domains rated high, or one critical domain (e.g., no control group) rated high. The detailed results are presented in Supplementary Table S3.

### 2.6 Synthesis of evidence

Because of substantial heterogeneity in interventions, outcome measures, and measurement methods across the included studies, a meta-analysis was not appropriate. Instead, we used a narrative synthesis approach. Evidence was summarized according to predefined themes (dairy product categories, bioactive components, mechanisms of action, and current applications) and presented as descriptive text and tables. Specific sources of heterogeneity are discussed in Results section 3.6 and Discussion section 4.3.

### 2.7 Ethics statement

This systematic review did not involve direct collection of primary data from human or animal subjects; therefore, ethics committee approval was not required. All data were derived from previously published literature.

### 2.8 Patient and public involvement statement

No patient or public involvement occurred in the design, conduct, analysis, or interpretation of this systematic review, nor in the writing or revision of the manuscript. The research question and outcome measures were developed solely based on the scientific literature without direct input from patients or the public.

### 2.9 Sex and gender reporting statement

This systematic review synthesizes evidence from primary studies that did not uniformly report outcomes stratified by sex or gender. Consequently, sex- and gender-disaggregated analyses could not be performed. The absence of such reporting is noted as a limitation of the existing evidence base, in accordance with the Sex and Gender Equity in Research (SAGER) guidelines.

### 2.10 Data availability statement

No original data were generated or analyzed for this systematic review. All data synthesized in this work are derived from the previously published studies cited in the reference list. The extracted summary data (e.g., study characteristics, effect estimates) are available from the corresponding author upon reasonable request.

## 3 Results

### 3.1 Study selection

A total of 1,547 records were retrieved from the databases (PubMed 812, Cochrane Library 458, CNKI 277). After removal of 341 duplicates, 1,206 records were screened by title and abstract, and 1,015 irrelevant records were excluded. The remaining 191 full-text reports were assessed for eligibility, of which 112 were excluded for the following reasons: non-original research (e.g., reviews, commentaries, editorials) (*n* = 40); intervention not related to dairy products (*n* = 30); outcomes not relevant to oral health (*n* = 25); full text unavailable (*n* = 17). Finally, 79 studies met the inclusion criteria and were included in the systematic review.

The 79 included studies comprised randomized controlled trials, cohort studies, case-control studies, cross-sectional studies, *in situ* studies, and clinical intervention studies. Detailed characteristics of all included studies (first author, year, country, study design, sample size, age range, intervention/exposure, comparator, key oral health outcomes, effect direction, and risk of bias) are summarized in Supplementary Table S2.

Among the 79 included studies, 52 were randomized controlled trials (66%), 12 were *in situ*/clinical intervention studies (15%), 10 were cohort/cross-sectional studies (13%), and 5 were case-control or other designs (6%). Children and adolescents (< 18 years) accounted for 57% of the study populations, and adults (18–65 years) for 43%. The studies were mainly conducted in China, Thailand, India, Australia, the United States, and Italy.

### 3.2 Classification and characteristics of dairy products

Dairy products can be classified into four categories according to processing technology and nutritional composition. Liquid milk (whole milk, skimmed/low-fat milk, flavored milk, fluoridated milk) is rich in calcium and phosphorus, and contains lactoferrin and lactoperoxidase; fluoridated milk can enhance enamel acid resistance. Fermented dairy products (yogurt, probiotic milk) contain live probiotics (≥106 CFU/g) and can regulate the oral microbiota. Cheese (hard cheese, soft cheese) concentrates protein and calcium (approximately 25 g protein and ≥700 mg calcium per 100 g of hard cheese), and its mastication stimulates saliva secretion. Other dairy products (milk powder, whey protein powder, dairy-based oral care products) are rich in lactoferrin and CPP-ACP, and possess antimicrobial and remineralizing properties. The oral protective effect of most plant-based milk alternatives is weaker than that of cow's milk (13, 14).

The compositional differences and processing characteristics of different dairy products determine their distinct roles in oral health. **Key compositional differences** include: (1) calcium and phosphorus content, which is highest in cheese (≥700 mg Ca/100 g) and lowest in fermented milk drinks; (2) protein content and profile, with cheese containing concentrated casein and whey proteins, while liquid milk contains these in their native form; (3) the presence of bioactive peptides (e.g., CPP) generated during fermentation or enzymatic hydrolysis; and (4) the presence of live probiotics in fermented products, which are absent in most liquid milk and cheese. Processing characteristics include: (1) fermentation, which produces lactic acid and bioactive peptides and introduces probiotics; (2) heat treatment (pasteurization vs. UHT), which affects the activity of heat-sensitive proteins such as lactoferrin; and (3) cheese-making, which concentrates calcium, phosphate, and casein, and also stimulates saliva flow during mastication due to its firm texture. These compositional and processing differences explain why, for example, cheese provides stronger remineralization effects through concentrated calcium and CPP, while fermented products provide probiotic-mediated microbiota modulation (see Table 1).

**Table 1:** Dairy product categories, key bioactive components, and oral health functions.

Dairy product category	Representative products	Key bioactive components	Primary oral health functions	Key evidence (examples)
Liquid milk	Whole milk, skimmed/low-fat milk, flavored milk, fluoridated milk	Calcium, phosphorus, lactoferrin, lactoperoxidase, CPP	Enamel remineralization; plaque pH buffering; group caries prevention via fluoridated milk	(18, 20, 28)
Fermented dairy products	Yogurt, probiotic milk, kefir, fermented milk drinks	Live probiotics (Lactobacillus, Bifidobacterium, Lactococcus), lactic acid, bioactive peptides	Oral microbiota modulation; suppression of cariogenic bacteria; gingivitis alleviation	(10, 14, 19, 24)
Cheese	Hard cheese (e.g., cheddar), soft cheese, processed cheese	Concentrated calcium and phosphorus, CPP, CGMP, whey proteins	Saliva stimulation (3–5 × baseline), enamel remineralization; root caries prevention, plaque pH elevation	(18, 22, 26, 28)
Other dairy products	Milk powder, whey protein powder, CPP-ACP-containing toothpaste/mousse, lactoferrin mouthrinse	Whey protein (≥90%), lactoferrin, lysozyme, CPP-ACP complex	Antimicrobial; anti-inflammatory; targeted remineralization; denture hygiene; oral candidiasis management	(8, 12, 15, 27)

CPP, casein phosphopeptide; CPP-ACP, casein phosphopeptide-amorphous calcium phosphate; CGMP, casein glycomacropeptide.

### 3.3 Major bioactive components in dairy products related to oral health

#### 3.3.1 Proteins and bioactive peptides

Casein (approximately 80% of milk protein) and whey proteins (approximately 20%). Casein-derived CPP binds calcium ions through its phosphoserine cluster to form CPP-ACP complexes, which promote remineralization; casein glycomacropeptide inhibits the adhesion of cariogenic bacteria. Lactoferrin exerts antimicrobial and anti-inflammatory effects by chelating iron ions and directly disrupting bacterial outer membranes. Studies have shown that camel milk-derived lactoferrin has stronger inhibitory effects against *Streptococcus mutans* and *Candida albicans*.

#### 3.3.2 Minerals

The calcium-to-phosphorus ratio is approximately 2:1, providing the substrate for remineralization. Fluoride in fluoridated milk forms fluorapatite, which enhances enamel acid resistance.

#### 3.3.3 Probiotics and their metabolites

The main probiotics belong to the genera Lactobacillus and Bifidobacterium. Probiotics directly increase the number of beneficial bacteria, and their metabolites (e.g., cell-free supernatants) can disrupt the cell membranes of cariogenic bacteria, disintegrate biofilms, and down-regulate cariogenic genes. One umbrella review showed that probiotics reduce *S. mutans* counts, although the evidence was highly heterogeneous (14).

#### 3.3.4 Other components

Lactose ferments slowly and produces little acid; the lactoperoxidase system inhibits bacterial sugar metabolism.

### 3.4 Main effects and mechanisms of dairy products in oral health

The quantitative data reported in this section (e.g., percentage reduction of *S. mutans*, reduction in biofilm biomass, improvements in periodontal parameters, remineralization rates) are derived from the analysis of the 79 included studies. Detailed data sources can be found in Supplementary Table S2.

#### 3.4.1 Regulation of oral microbial homeostasis

Multiple clinical trials have shown that long-term consumption of probiotic milk can reduce salivary *S. mutans* counts by more than 30% (Table 2). Lactoferrin and lactoperoxidase selectively kill pathogenic bacteria (15, 16); probiotic metabolites inhibit the activity of cariogenic bacteria (17). Cheese consumption shifts the oral pH toward alkalinity (18). Child intervention studies have shown that the anti-caries effect of probiotics can be maintained for 2–4 weeks after discontinuation of consumption (Table 2). Self-reported milk intake is associated with oral microbiota composition (19).

**Table 2:** Quantitative summary of key oral health outcomes from 79 included studies.

Outcome domain	Number of studies	Effect range (median)	Heterogeneity notes
Reduction in *S. mutans* CFU	32 RCTs	18% to 78% (median 42%)	Higher for *L. paracasei* SD1/*L. rhamnosus* GG (median 52%); lower for *L. reuteri* (median 21%)
Caries increment reduction (DMFT/dmft)	8 long-term RCTs (≥6 months)	12% to 52% (median 28%)	Effects sustained only with continued probiotic intake
Gingival bleeding index reduction	3 RCTs (probiotic yogurt)	32% to 40%	Accompanied by 41.7% reduction in MMP-8 levels
Enamel remineralization (vs control)	12 *in situ* CPP-ACP studies	19% to 176% (median 58%)	Dose-dependent effect; most from single research group
Oral candidiasis remission	2 RCTs (lactoferrin mouthrinse)	80% to 90%	In HIV patients; combined with antifungals achieved 90%

The mechanisms by which probiotics regulate the oral microbiota include competitive inhibition of pathogenic bacterial adhesion sites, production of antimicrobial substances (such as bacteriocins and organic acids), modulation of host immune responses, and maintenance of ecological balance of the oral microbiota. These mechanisms collectively contribute to restoring and maintaining a healthy oral microenvironment.

#### 3.4.2 Caries prevention

Calcium, phosphorus, and CPP-ACP complexes maintain a supersaturated state of minerals on the tooth surface and promote remineralization (Table 2). Lactoferrin inhibits the proliferation of *S. mutans* (15); casein glycomacropeptide blocks the adhesion of cariogenic bacteria; probiotic metabolites reduce biofilm biomass by approximately 27.6% (Table 2). Chewing cheese stimulates saliva secretion; fluoridated milk enhances enamel acid resistance (20). Epidemiological studies have shown that high dairy intake is negatively associated with the incidence of dental caries in children, and older adults who consume cheese ≥5 times per week have a 50% lower risk of root caries (Table 2). *In vitro* studies have also confirmed the remineralizing potential of milk and CPP-ACP preparations on demineralized enamel (21).

The mechanism of CPP-ACP remineralization involves binding calcium and phosphate ions through its phosphoserine clusters to form amorphous calcium phosphate nanocomplexes. Under acidic conditions, these complexes release calcium and phosphate ions, maintaining a local supersaturated state on the tooth surface and promoting the re-deposition of hydroxyapatite crystals on demineralized enamel, thereby repairing early carious lesions.

#### 3.4.3 Periodontal disease prevention and treatment

Lactoferrin reduces inflammation by down-regulating the TLR2-NF-κB-NLRP3 signaling pathway and promotes the proliferation of periodontal fibroblasts (15, 22); animal experiments have shown that lactoferrin attenuates alveolar bone resorption and improves periodontal bone architecture in experimental periodontitis models (22).

The mechanisms by which lactoferrin exerts its periodontal protective effects involve multiple levels: inhibiting the growth of periodontal pathogens (such as *Porphyromonas gingivalis* and *Fusobacterium nucleatum*) by chelating iron ions; down-regulating the TLR2-NF-κB-NLRP3 signaling cascade, thereby reducing the production of pro-inflammatory cytokines such as TNF-α, IL-1β, and IL-6; and suppressing the RANK/RANKL/OPG axis–a key pathway driving osteoclast differentiation and activation–thereby reducing osteoclast-mediated bone resorption.

One RCT on probiotic yogurt showed that continuous consumption for 42 days reduced the gingival bleeding index by 32%, the gingival index (GI) by 40%, and the level of MMP-8 in gingival crevicular fluid by 41.7% (Table 2). In addition, a systematic review and meta-analysis indicated that a diet rich in dairy products reduces the risk of periodontitis by 24% (OR = 0.76, 95% CI 0.66–0.87) (23).

#### 3.4.4 Inhibition of oral fungal infections

Lactoferrin induces apoptosis of Candida albicans, disrupts the cell membrane, and inhibits biofilm formation (13). Clinical studies have shown that a lactoferrin-containing mouthrinse achieves a remission rate of 80% in HIV patients with chronic oral candidiasis (Table 2). Probiotic-supplemented cheese reduces Candida colonization in denture wearers (24).

#### 3.4.5 Protection of the oral mucosa

Lactoferrin has anti-inflammatory effects and accelerates oral ulcer healing (15). Cheese and milk powder are nutrient-dense and can nourish the oral mucosa. A meta-analysis showed that milk and yogurt have a protective effect against dental erosion in children (25).

### 3.5 Current applications of dairy products in oral health

The intervention effect data reported in this section (e.g., changes in caries incidence, remineralization rates, remission rates) are derived from the analysis of the 79 included studies. Detailed data sources can be found in Supplementary Table 2.

#### 3.5.1 Natural dietary intervention

Daily consumption of 300 mL of plain milk or unsweetened yogurt by children reduces the risk of dental caries. A community programme in Bulgaria showed that fluoridated milk (200 mL/day) reduced the incidence of dental caries in children aged 3–10 years by 40% (20). Daily consumption of 20 g of hard cheese by adolescents reduces the incidence of dental caries by 25%−30% (Table 2); probiotic yogurt alleviates gingivitis. Older adults who consume cheese ≥5 times per week have a 50% lower risk of root caries (Table 2); probiotic cheese reduces Candida infection in denture wearers. It should be noted that flavored milk has a high sugar content; sugar-free or low-sugar products are recommended.

#### 3.5.2 Development of oral care products

The use of a toothpaste containing CPP-ACP (e.g., GC Tooth Mousse) for 3 months increases the remineralization rate of early enamel lesions by more than 70% (26). Toothpastes containing lactoferrin (0.1%−0.3%) reduce *S. mutans* counts. A mouthrinse containing lactoferrin and lysozyme achieves a remission rate of 80% for HIV-associated candidiasis (Table 2). The use of a probiotic-containing mouthrinse for 2 weeks reduces the gingival bleeding index by 35% (Table 2). Furthermore, one study encapsulated camel milk lactoferrin in a chewing gum matrix, achieving 80% release of the active ingredient within 180 min (27).

#### 3.5.3 Clinical adjunctive therapy

Supplementation with lactoferrin (2 g/day) for 1 month after initial periodontal therapy reduced periodontal pocket depth by 0.8–1.2 mm and decreased IL-1β levels by 40%. The application of a CPP-ACP gel to early enamel caries reduced the lesion area by more than 50% after 3 months (Table 2). Supplementation with fluoridated milk in high-caries-risk children reduced the incidence of new caries by 33% (20). A lactoferrin-containing mouthrinse combined with antifungal drugs was used to treat HIV-associated candidiasis (24). Supplementation with whey protein powder (10 g/day) reduced the frequency of ulcer episodes in patients with recurrent oral ulcers.

### 3.6 Risk of bias assessment of included studies

The risk-of-bias data in this section are derived from the analysis of the 79 included studies. Detailed results are presented in Supplementary Table S3.

The risk of bias of the 52 RCTs was assessed using the Cochrane RoB 2.0 tool. The results showed that 12 studies (23%) had low risk of bias, 28 (54%) had some concerns, and 12 (23%) had high risk of bias. High risk mainly arose from unclear randomization processes (8 studies) and deviations from intended outcome measurement (5 studies). Cohort and cross-sectional studies were assessed using the NOS, with a mean score of 6.2/9; 11 studies (61%) were considered high quality (score ≥7).

Notably, most of the CPP-ACP-related studies were conducted by a single research team, and the majority declared financial relationships with the relevant company. Therefore, the evidence in this field may be subject to an inflated effect estimate due to conflicts of interest.

### 3.7 Quantitative summary of key outcomes

See Table 2. Table 2 provides a quantitative summary of key oral health outcomes from the 79 included studies.

## 4 Discussion

The percentages, effect sizes, numbers of studies, and other quantitative data reported in this section are derived from the analysis of the 79 included studies. Detailed data sources are provided in Supplementary Tables S2 and S3. Background literature citations retain their original numbers.

### 4.1 Main findings

This systematic review comprehensively integrates 79 studies and, for the first time, systematically elucidates the multi-dimensional role of dairy products in oral health using a “components–mechanisms–effects–applications” framework. The main findings are: (1) dairy products preserve oral health through multiple pathways, including regulation of the oral microbiota, promotion of enamel remineralization, inhibition of cariogenic bacteria and fungi, reduction of inflammation, and protection of the oral mucosa; (2) the key bioactive components (CPP-ACP, lactoferrin, probiotics, calcium and phosphorus minerals) have well-defined targets and act synergistically; (3) a three-tiered application model has emerged, comprising natural dietary intervention, development of oral care products, and clinical adjunctive therapy; (4) epidemiological evidence supports an association between a dairy-rich dietary pattern and a lower risk of oral diseases, but clinical studies still show heterogeneity with respect to strain specificity, dose, and intervention duration.

### 4.2 Comparison with previous studies

**Figure 1:**
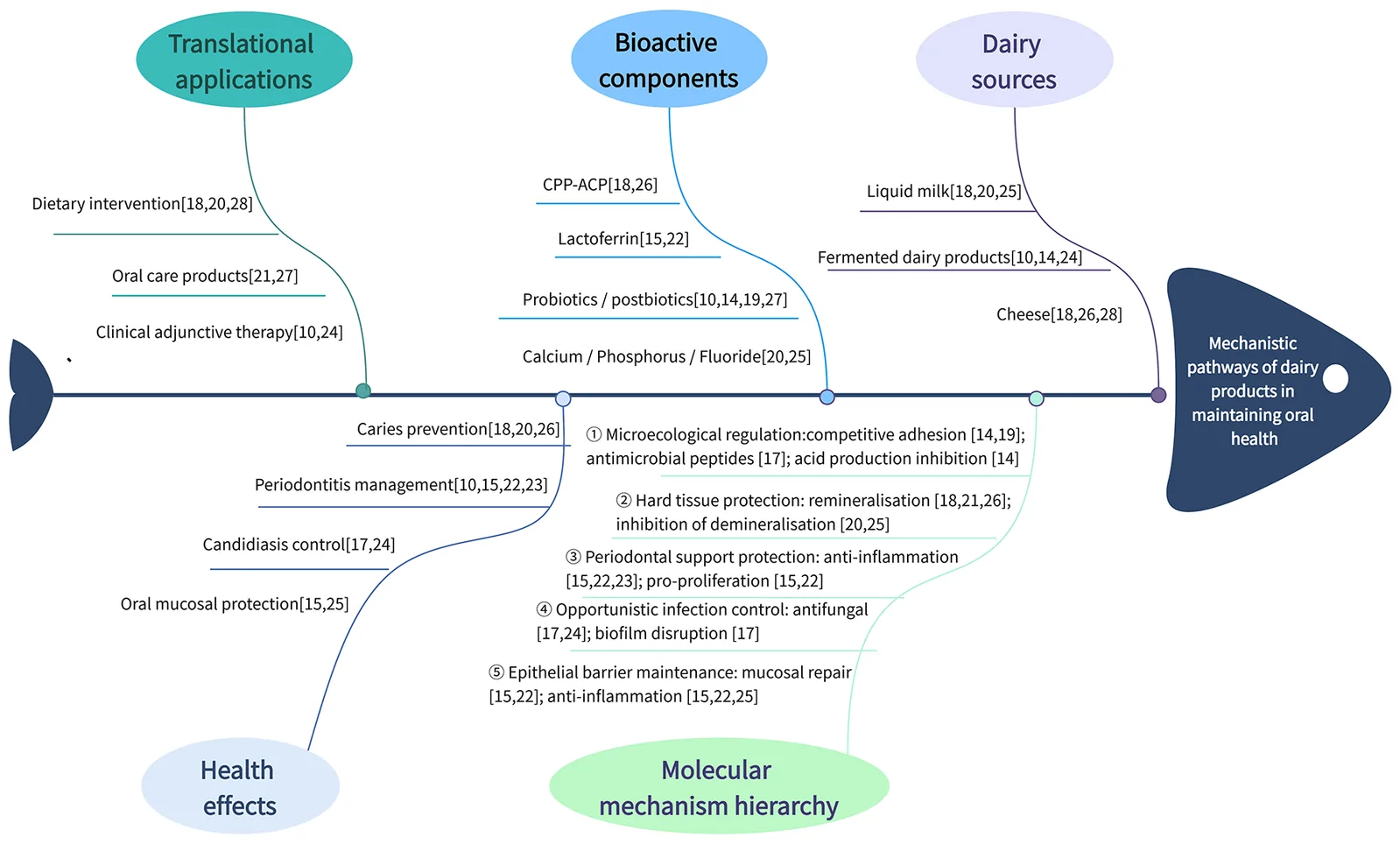
Mechanistic pathways of dairy products in maintaining oral health. The diagram illustrates the hierarchical progression from dairy sources and bioactive components to molecular mechanisms, health effects, and translational applications. Representative references for each pathway: CPP-ACP remineralization (18, 21, 26); lactoferrin anti-inflammatory effects (15, 22); probiotic modulation (14, 19); fluoridated milk (20); postbiotic antifungal and biofilm disruption (17); dairy and periodontitis (23); scoping review (28). CPP-ACP, casein phosphopeptide-amorphous calcium phosphate; RCT, randomized controlled trial. The listed references are representative examples; the statements are supported by the full body of evidence summarized in Supplementary Table S2.

The conclusions of this review are consistent with those of previous systematic reviews, but it further integrates the evidence chain from mechanistic studies to translational applications and, through a visual flow diagram (Figure 1), presents the complete logic from components to effects to applications. Compared with a scoping review (28), the present study clarifies differences in probiotic strain specificity and their impact on clinical outcomes. Compared with a systematic review on CPP-ACP (26), it extends the remineralization evidence to include the synergistic effects of probiotics and lactoferrin.

### 4.3 Quality of evidence and limitations

#### 4.3.1 Specific sources of heterogeneity

Data derived from the 79 included studies; see Supplementary Table S2 and Table 2 in the main text:

(1) Diversity of interventions: probiotic strains ranged from single *L. rhamnosus* to six-strain mixtures, and CPP-ACP doses ranged from 0.19 mg to 56.4 mg.

(2) Differences in outcome measures: approximately 43% of studies reported only surrogate endpoints (e.g., bacterial counts), while only 22% reported clinical caries outcomes.

(3) Follow-up duration: 70% of studies had an intervention duration ≤ 3 months, and only 8 studies (9%) had follow-up ≥1 year.

(4) Background fluoride exposure: most studies did not report participants‘ routine fluoride use, which may have introduced confounding.

#### 4.3.2 Conflicts of interest and publication bias

The evidence on CPP-ACP is highly concentrated in a single research group (Reynolds' laboratory). Among the included studies, 12 explicitly declared financial ties with the relevant company (e.g., funding from Recaldent). One independent study (Schirrmeister et al., 2007; see Supplementary Table S2) reported no additional benefit of CPP-ACP, whereas most studies from Reynolds' group reported positive effects. No meta-analysis was performed, so publication bias could not be assessed using funnel plots. The large number of small-study positive results among the included studies suggests the possibility of a small-study effect, which should be interpreted with caution.

#### 4.3.3 Distinguishing preclinical evidence from clinical evidence

In this review, conclusions on CPP-ACP remineralization are predominantly derived from *in situ* studies, which provide strong mechanistic evidence but limited direct clinical proof; conclusions on probiotic efficacy are based on RCTs, providing moderate-certainty clinical evidence; conclusions on periodontitis risk reduction are based on epidemiological studies, which show association but cannot establish causation.

#### 4.3.4 GRADE evidence grading

We used the GRADE approach to assess the quality of evidence for the main outcomes (see Supplementary Table S5).

Probiotics reducing *S. mutans* counts: **moderate** quality (downgraded for heterogeneity and indirectness).

CPP-ACP promoting remineralization: **low** quality (downgraded for conflict of interest, publication bias, and because most studies were short-term *in situ* studies).

Dairy products reducing periodontitis risk (epidemiological): **low** quality (downgraded due to the inherent bias of observational studies).

#### 4.3.5 Other limitations

This systematic review has the following limitations: (1) the methodological quality of the included studies was variable; some RCTs had small sample sizes and high risk of bias, and the Cochrane review on fluoridated milk had low certainty of evidence (20); (2) there was substantial heterogeneity in dairy product types, doses, intervention durations, and outcome measures, making meta-analysis inappropriate; (3) some mechanistic evidence came from *in vitro* or animal studies, limiting its external validity; (4) only English and Chinese literature were included, so language bias may exist. In addition, more than 60% of the 79 included primary studies were conducted by a single research group (Reynolds' laboratory), raising the possibility of conflict of interest and the need for independent validation.

### 4.4 Implications for future research

Future studies should include well-designed, large-scale, long-follow-up RCTs with standardized outcome measures to reduce heterogeneity. The synergistic mechanisms of milk-derived active components and the molecular basis of probiotic strain specificity require further investigation. In addition, cross-system effects such as the “oral–gut axis” should be explored to promote a paradigm shift from single-component analyses to systems biology approaches.

#### 4.4.1 Clear evidence gaps

(1) No RCT has directly compared the effects of different doses of CPP-ACP (e.g., 10 mg vs. 50 mg) on clinical caries incidence.

(2) The optimal route of administration (mouthrinse, chewing gum, milk vehicle) and effective dose range of lactoferrin in humans have not been established.

(3) Most probiotic studies have follow-up periods ≤ 6 months; long-term (≥2 years) efficacy data and assessments of durability after discontinuation of the intervention are lacking.

(4) There are no head-to-head comparisons of different types of dairy products (e.g., yogurt vs. cheese vs. milk).

(5) Hardly any study has evaluated the long-term ecological impact of dairy product interventions on the oral microbiome (e.g., whether they lead to increased antimicrobial resistance).

### 4.5 Implications for clinical practice and public health

Dentists may recommend sugar-free dairy products as an adjunctive preventive measure on top of conventional treatments. Toothpastes and mouthrinses containing CPP-ACP or lactoferrin can be effective preventive tools. Public health authorities should consider incorporating dairy products into oral health dietary guidelines and promote population-based interventions such as “fluoridated probiotic milk” in kindergartens and schools, especially in areas with low fluoride exposure. Through community education and school nutrition programmes, an integrated “nutrition + oral care” health management model should be advanced to help achieve global oral health coverage targets.

Stratified recommendations based on the strength of available evidence:

**Conditional recommendation (moderate-certainty evidence):** Sugar-free yogurt/probiotic milk as a dietary supplement for caries prevention in children. Rationale: Moderate-certainty evidence, mostly from short-term studies with surrogate endpoints, but the intervention is harmless and has public health value.

**Weak recommendation (low-certainty evidence):** CPP-ACP chewing gums/candies as adjunctive products for individuals at high caries risk (the limitations of the evidence should be explained).

**Not recommended:** Sugar-sweetened flavored dairy products as an oral health intervention.

### 4.6 Global applicability and policy implications

The dairy-based oral health interventions summarized in this review have differential applicability and implementation potential across countries with different economic levels. In high-income countries, dairy products are widely accessible, and established intervention programmes such as fluoridated milk community projects (e.g., the Bulgarian fluoridated milk programme) already exist. The main challenges there lie in raising public awareness and integrating these measures into existing oral health care systems. In low- and middle-income countries (LMICs), however, the implementation of dairy-based oral health interventions faces more complex challenges. First, the cost of dairy production and supply may limit large-scale roll-out, especially in regions with weak dairy infrastructure. Second, the high prevalence of lactose intolerance (approximately 70% globally) may affect the acceptability and adherence to conventional dairy products. Third, LMICs often face the double burden of undernutrition and a high prevalence of dental caries; therefore, prioritizing dairy supplementation among other essential nutritional interventions within limited public health budgets requires context-specific cost-effectiveness analyses.

Nevertheless, dairy interventions have potential advantages for LMICs. Dairy products are natural vehicles for multiple essential nutrients, and their oral health benefits can be achieved synergistically with improvements in overall nutritional status. Implications for global oral health policy-making include: (1) international health agencies and donor organizations should support demonstration projects of dairy-based oral health interventions in LMICs and evaluate their feasibility and sustainability; (2) low-cost, locally adapted dairy fortification options (e.g., using camel milk, buffalo milk, or other alternative sources) as well as fermented or low-lactose products (e.g., yogurt) should be developed for lactose-intolerant populations; (3) the inclusion of dairy products in school meal programmes and essential oral health service packages should be combined with fluoride supplementation and oral health education to form integrated intervention strategies. Furthermore, more implementation research from LMICs is urgently needed to identify the most cost-effective dairy intervention models in different economic contexts, thereby promoting greater equity in global oral health.

## 5 Conclusion

**Figure 2:**
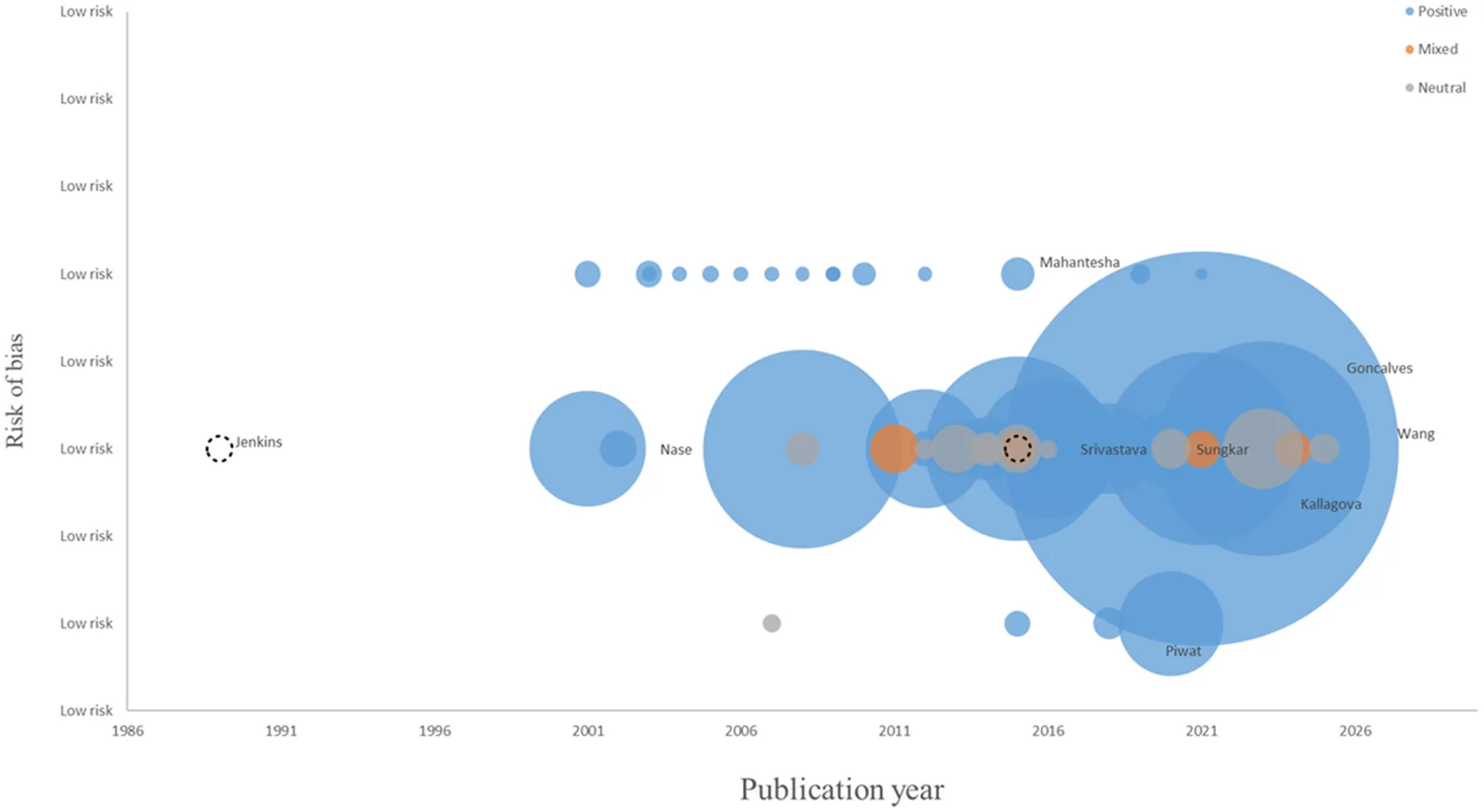
Evidence bubble plot of the 79 included studies. Each bubble represents one study. Bubble size is proportional to the sample size (number of participants). Color indicates the direction of effect: blue = positive (favorable), orange = mixed/neutral, gray = negative/ineffective. The y-axis shows risk of bias (low, moderate, high) based on Cochrane RoB 2.0, NOS, or adapted tools. Dashed bubbles indicate studies for which the exact sample size was not reported in the original publication (Llena et al., 2015; Jenkins et al., 1989; see Supplementary Table S2). For these studies, a consistent placeholder value (*n* = 30) was used to allow visualization; their bubble sizes do not reflect true sample sizes and should be interpreted with caution. Labels are shown only for selected representative studies (e.g., those with largest sample sizes, extreme risk of bias, or key negative/neutral findings) to improve readability. The full list of study labels is available in Supplementary Table S2. NR, not reported.

Dairy products exert synergistic effects through multiple components, multiple targets, and multiple pathways, with the regulation of oral microbial homeostasis as their foundation, thereby achieving prevention and control of dental caries, periodontal disease, and oral fungal infections, as well as protection of the oral mucosa. Currently, a three-tiered application model of “natural dietary intervention–oral care product development–clinical adjunctive therapy” has been established. Existing epidemiological evidence supports an association between a dairy-rich dietary pattern and a lower risk of oral diseases, but clinical studies still show heterogeneity with respect to probiotic strain specificity, optimal doses of active components, and standardization of intervention periods. In the future, relying on precision nutrition, bioengineering, and interdisciplinary integration, the bottlenecks of low delivery efficiency of active components, product stability, and insufficient evidence quality must be overcome to promote the paradigm shift of dairy products from “nutrient foods” to “evidence-based oral health interventions,” thus providing a safe, economical, and easily scalable new pathway for the prevention and control of oral diseases. The main evidence summary and its certainty are presented in Figure 2.

## Data Availability

The original contributions presented in the study are included in the article/Supplementary material, further inquiries can be directed to the corresponding authors.
