# Supplementary material for: Functional mechanisms, current applications, and future perspectives of dairy products in oral health
Source: Front Public Health. 2026 Aug 31;14:1898007. doi: 10.3389/fpubh.2026.1898007 (PMC13572671; doi:10.3389/fpubh.2026.1898007)
Supplement: Supplementary file 1 [file Table_1.docx]

**Table S1. Complete search strategies used in PubMed, Cochrane Library, and CNKI (search date: 15 April 2026)**

| **Database** | **Search string** | **Filters** | **Records retrieved (n)** |
| --- | --- | --- | --- |
| **PubMed** | ("dairy products"[MeSH Terms] OR "dairy products"[Title/Abstract] OR "milk"[Title/Abstract] OR "yogurt"[Title/Abstract] OR "cheese"[Title/Abstract] OR "whey protein"[Title/Abstract] OR "lactoferrin"[Title/Abstract] OR "casein phosphopeptide"[Title/Abstract] OR "probiotics"[Title/Abstract]) AND ("oral health"[MeSH Terms] OR "oral health"[Title/Abstract] OR "dental caries"[Title/Abstract] OR "periodontitis"[Title/Abstract] OR "candidiasis"[Title/Abstract] OR "oral microbiota"[Title/Abstract] OR "enamel remineralization"[Title/Abstract]) | 1989–2026; English | 812 |
| **Cochrane Library** | ("dairy products" OR "milk" OR "yogurt" OR "cheese" OR "whey protein" OR "lactoferrin" OR "casein phosphopeptide" OR "probiotics") AND ("oral health" OR "dental caries" OR "periodontitis" OR "candidiasis" OR "oral microbiota" OR "enamel remineralization") | 1989–2026 | 458 |
| **CNKI*** | SU=(‘乳制品’+‘牛奶’+‘酸奶’+‘奶酪’+‘乳清蛋白’+‘乳铁蛋白’+‘酪蛋白磷酸肽’+‘益生菌’) AND SU=(‘口腔健康’+‘龋齿’+‘牙周炎’+‘口腔念珠菌病’+‘口腔微生态’+‘牙釉质再矿化’) | 1989–2026; Chinese | 277 |

*The search string shown is the actual Chinese syntax used in the CNKI database; no English translation is provided.
