# Supplementary material for: Functional mechanisms, current applications, and future perspectives of dairy products in oral health
Source: Front Public Health. 2026 Aug 31;14:1898007. doi: 10.3389/fpubh.2026.1898007 (PMC13572671; doi:10.3389/fpubh.2026.1898007)
Supplement: Supplementary file 2 [file Table_2.docx]

**Table S2-Study characteristics**

| **ID** | **First author** | **year** | **Country** | **Study design** | **Sample size** | **Age range** | **Intervention** | **Comparator** | **Key oral health outcomes** | **Effect direction** | **Risk of bias** | **DOI** |
| --- | --- | --- | --- | --- | --- | --- | --- | --- | --- | --- | --- | --- |
| 1 | Nase L | 2001 | Finland | RCT | 594 | 1-6 years | *L. rhamnosus* GG milk | Placebo milk | Caries increment↓ | Positive | Moderate (JADAD 3) | 10.1159/000047484 |
| 2 | Shen P | 2001 | Australia | In situ | 30 | 31.5 years (mean) | CPP-ACP gum (0.19-56.4 mg) | No CPP-ACP gum | Enamel remineralization (9-152%) | Positive | High (COI) | 10.1177/00220345010800120801 |
| 3 | Ahola AJ | 2002 | Finland | RCT | 60 | Adults (mean NR) | Probiotic cheese *(L. rhamnosus* GG) | Regular cheese | Salivary *S. mutans*, yeast↓ | Positive | Moderate | 10.1016/S0003-9969(02)00112-7 |
| 4 | Cai F | 2003 | Australia | In situ | 10 | 34 years (mean) | CPP-ACP candy (18.8-56.4 mg) | No candy | Enamel remineralization (12.5-19.4%) | Positive | High (COI) | doi:10.1111/j.1834-7819.2003.tb00037.x |
| 5 | Reynolds EC | 2003 | Australia | Clinical | 30 | 33 years (mean) | CPP-ACP gum (9.5 mg) | No gum | Plaque Ca, phosphate retention | Positive | High (COI) | 10.1177/154405910308200311 |
| 6 | Iijima Y | 2004 | Australia | In situ | 10 | 33 years (mean) | CPP-ACP gum (18.8 mg) | No CPP-ACP gum | Enamel remineralization (17% vs 9%), acid resistance­ | Positive | High (COI) | 10.1159/000080585 |
| 7 | Itthagarun A | 2005 | Canada | In situ | 12 | 33.5 years (mean) | CPP-ACP + dicalcium phosphate gum | No gum | Enamel remineralization (65% vs 10-52%) | Positive | High (COI) | 10.1159/000084806 |
| 8 | Cai F | 2007 | Australia | In situ | 10 | 34.5 years (mean) | CPP-ACP + citric acid gum (18.8 mg) | Citric acid only gum | Enamel remineralization (13% vs 2.6%) | Positive | High (COI) | 10.1159/000104796 |
| 9 | Schirrmeister JF | 2007 | Germany | In situ | 15 | 27.5 years (mean) | CPP-ACP gum (13 mg) | Calcium gum / no gum | Enamel remineralization (no benefit) | Neutral | Low (independent) | 10.1159/000098043 |
| 10 | Caglar E | 2008 | Turkey | RCT | 52 | 8-10 years | *B. bifidum* DN-173 010 yogurt | No yogurt | No effect on dental plaque | Neutral | Moderate | 10.1111/j.1365-263X.2007.00866.x |
| 11 | Manton DJ | 2008 | Australia | In situ | 10 | NR (adults) | CPP-ACP gum (Trident White) | Xylitol gums (Orbit) | Enamel remineralization (18% vs 8-10%) | Positive | High (COI) | 10.1111/j.1365-263X.2008.00920.x |
| 12 | Morgan MV | 2008 | Australia | In vivo RCT (2y) | 1749 | 12 years (mean) | CPP-ACP gum (54 mg) | Sorbitol gum | Proximal caries progression↓(18% reduction) | Positive | Moderate | 10.1159/000128561 |
| 13 | Cai F | 2009 | Australia | In situ | 10 | 40 years (mean) | CPP-ACP gum (3.5 mg / 0.4%) | Other sugar-free gums (Orbit/Extra) | Enamel remineralization (20% vs 9-12%) | Positive | High (COI) | 10.1016/j.jdent.2009.06.003 |
| 14 | Walker GD | 2009 | Australia | In situ | 10 | 39 years (mean) | CPP-ACP milk (2.0-5.0 g/L) | Plain milk | Enamel remineralization (5.4-7.9% vs 3.0%) | Positive | High (COI) | 10.1111/j.1834-7819.2009.01127.x |
| 15 | Tanaka K | 2012 | Japan | Prospective cohort | 315 mother-child pairs | 41–50 months (~3.5–4 years) | Cheese intake (highest vs. lowest tertile) Dairy products (highest vs. lowest tertile) Calcium intake (highest vs. lowest tertile) | Lowest tertile group | Children with ≥1 primary tooth decayed, missing, or filled (dmft >0) | Positive | Moderate | 10.1186/1475-2891-11-33 |
| 16 | Walker GD | 2010 | Australia | In situ | 24 | 34-35 years (mean) | CPP-ACP candy (0.5-1% ) | Sugar / sugar-free candy | Enamel remineralization (-6%→+12.7%) | Positive | High (COI) | 10.1159/000275572 |
| 17 | Aminabadi NA | 2011 | Iran | RCT | 105 | 6-9 years | *L. rhamnosus* GG yogurt ± CHX | CHX only / probiotic only | *S. mutans* (mixed) | Mixed | Moderate | 10.1159/000325741 |
| 18 | Cildir SK | 2012 | Turkey | RCT, crossover | 19 | 4-12 years | *L. reuteri* drops (DSM 17938 + ATCC PTA 5289) | Placebo | *S. mutans*, *Lactobacillus* (not significant) | Neutral | Moderate | 10.1597/10-035 |
| 19 | Cochrane NJ | 2012 | Australia | In situ | 9 | 36.5 years (mean) | CPP-ACP gum (18.8 mg) | No gum / gum without CPP-ACP | Greater remineralization than control | Positive | High (COI) | 10.1159/000337240 |
| 20 | Iniesta M | 2012 | Spain | RCT | 54 | University students | Probiotic yogurt (*L. reuteri*) | Placebo yogurt | Gingival bleeding index↓32%, GI↓40%, MMP-8↓41.7% | Positive | Moderate | 10.1111/j.1600-051X.2012.01914.x |
| 21 | Campus G | 2014 | Italy | RCT | 181 | 6-8 years | *L. brevis* CD2 lozenge | Placebo | Plaque pH (AUC)↓,*S. mutans*↓*,* Bleeding↓ | Positive | Moderate | 10.1007/s00784-013-0980-9 |
| 22 | Alp S | 2018 | Turkey | RCT | 45 (15 per group) | 14.1–14.9 years (mean) | Kefir (200 mL/day) /Probiotic toothpaste | No probiotic treatment | ↓Salivary *S. mutans* and *Lactobacillus* (P<0.05) | Positive | Low | 10.1016/j.ajodo.2018.01.010 |
| 23 | Bhalla M | 2015 | India | RCT | 30 | 12-14 years | *B. lactis* Bb12 curd | Not specified | *S. mutans*↓ | Positive | Low (JADAD 2) | 10.4103/2231-0762.151970 |
| 24 | Chinnappa A | 2013 | India | RCT | 40 | 12-14 years | *B. lactis* Bb12 + *L. acidophilus* La-5 ice cream | Not specified | *S. mutans*↓ | Positive | Moderate | 10.4103/0970-9290.123363 |
| 25 | Llena C | 2015 | Spain | RCT | NR (786 lesions) | 6–14 years | CPP-ACP / CCP-ACFP toothpaste / fluoride varnish | Placebo | Early caries remineralization | Positive | High (PLoS ONE 2018) | PMID: 26418918 |
| 26 | Nozari A | 2015 | Iran | RCT | 49 | 6-12 years | *B. lactis* probiotic yogurt | Normal yogurt | *S. mutans* (probiotic NS; normal yogurt sig.) | Mixed | Moderate | PMID: 26046102 |
| 27 | Piwat S | 2020 | Thailand | RCT | 487 | 3.2±0.8 years | *L. paracasei* SD1 milk (daily or triweekly) | Placebo milk | Caries regression, new caries↓ | Positive | Low (RoB 2.0) | 10.1159/000509926 |
| 28 | Srivastava S | 2016 | India | RCT | 60 | 20-25 years | *L. acidophilus* curd | Placebo | Salivary pH↑, *S. mutans*↓ | Positive | Moderate | 10.7860/JCDR/2016/15530.7178 |
| 29 | Zaki NAA | 2015 | NR | Observational | 60 | 2-6 years | Dietary intake (including dairy) | None | Caries risk↓ | Positive | Moderate (NIH fair) | 10.1111/ipd.12150 |
| 30 | Hegde A | 2014 | India (est.) | Case-control | 90 | 5 years | Milk, cheese, CPP-ACP mousse | Caries-resistant children | Salivary Ca, phosphate↑ | Positive | Moderate | 10.17796/jcpd.38.4.l172u301208153v2 |
| 31 | Neves PAM | 2016 | NR | Observational | 16 | >24 months | Breast milk | None | No decrease in biofilm pH | Neutral | Moderate | 10.1159/000445910 |
| 32 | Nirunsittirat A | 2016 | NR | Prospective cohort | 860 | 21 days-36 months | Breastfeeding duration | None | Caries prevention (≤11 months) | Positive | Moderate | 10.1159/000448145 |
| 33 | Johansson I | 2018 | Sweden | Prospective cohort | 154 | 17 years | Self-reported milk intake | None | Oral microbiome, caries-associated bacteria↓ | Positive | Moderate | 10.1371/journal.pone.0193504 |
| 34 | Sungkar S | 2020 | Indonesia | Observational | 22 | 10-12 years | Cheese vs. milk | None | Salivary buffer capacity (cheese better) | Positive | Moderate | 10.4028/www.scientific.net/JBBBE.48.105 |
| 35 | Wang J | 2021 | USA | Observational (NHANES) | 6885 | 2-17 years | Whole/low-fat/skim milk, yogurt, cheese, cream | None | High yogurt, low cheese associated with lower caries risk | Positive | Moderate (NIH good) | 10.6133/apjcn.202106_30(2).0013 |
| 36 | Garcia-Pola M | 2021 | Spain | Observational | 166 | 6 years | Milk and dairy intake | None | Protective effect | Positive | Moderate | 10.1922/CDH_00056GarciaPola07 |
| 37 | Yardimci H | 2021 | NR | Cross-sectional | 153 | 30-71 months | Milk and dairy intake | None | Positive effect on dental health | Positive | Moderate | 10.1177/2158244021997413 |
| 38 | Olczak-Kowalczyk D | 2021 | Poland | Observational | 1638 | 3 years | Unsweetened milk | Sweetened beverages | Caries risk↓ | Positive | Moderate | 10.3389/FPED.2021.583660 |
| 39 | Pinto GS | 2014 | NR | RCT | 30 | 15 years | *B. animalis* subsp. *lactis* yogurt | Placebo | No additional benefit | Neutral | Moderate | 10.1159/000353467 |
| 40 | Caglar E | 2014 | Turkey | RCT | 52 | 8-10 years | *B. bifidum* DN-173 010 yogurt | None | No effect on dental plaque | Neutral | Moderate | 10.17796/jcpd.38.4.p608312353256684 |
| 41 | Mahantesha T | 2015 | India | Pre-post (no control) | 50 | 6-12 years | Probiotic ice cream vs. drink | None (pre-post) | *S. mutans*↓(ice cream better) | Positive | High (no control) | PMID: 26435616 |
| 42 | Ashwin D | 2015 | India | RCT | 60 | 6-12 years | *B. lactis* Bb-12 + *L. acidophilus* La-5 ice cream | Placebo | *S. mutans*↓at 7d,30d; not at 6m | Mixed | Moderate | 10.7860/JCDR/2015/10942.5532 |
| 43 | Lodi CS | 2015 | NR | RCT | 10 | NR | Probiotic fermented milk | None | Oral microorganisms↓ | Positive | Moderate | 10.1590/1807-3107BOR-2015.vol29.0033 |
| 44 | Villavicencio J | 2018 | NR | Cluster RCT | 363 | 3-4 years | *L. rhamnosus* + *B. longum* milk | Placebo | Lactobacillus↓, salivary buffer capacity↑ | Positive | Moderate | 10.1590/1678-7757-2017-0318 |
| 45 | Patil RU | 2019 | India | RCT | 10 | 7-12 years | Multi-strain probiotic milk (paste) | Not specified | *S. mutans*↓ | Positive | Moderate | 10.4103/JISPPD.JISPPD_519_20 |
| 46 | Sandoval F | 2021 | Chile | RCT | 42 | 2-3 years | *L. rhamnosus* SP1 milk | Standard milk | Caries occurrence↓,salivary hβD-3↓ | Positive | Moderate | 10.1007/s00784-020-03712-8 |
| 47 | Xu L | 2021 | China | Pre-post | 6 | 49.3 months (mean) | Probiotic yogurt | None (pre-post) | Altered salivary microbiota | Positive | High (no control) | 10.1007/S00203-021-02292-9 |
| 48 | Reddy S | 2021 | India | Interventional | 80 | 8-12 years | Kefir milk, probiotic curd, probiotic drink | Control | *S. mutans*↓(kefir and curd) | Positive | Moderate | 10.5005/jp-journals-10005-1883 |
| 49 | Sakhare S | 2021 | India | RCT | 62 | 6-12 years | *L. acidophilus* La-5 + *B. lactis* Bb12 mouth rinse | Control | *S. mutans*↓(24d); no pH change | Mixed | Moderate | 10.4103/jisppd.jisppd_236_21 |
| 50 | Guru Vishnu C | 2023 | India | RCT | 20 | 3-6 years | Probiotic curd | No extra supplement | ↓Dental plaque pH; ↓S. mutans count | Positive | Moderate | 10.47750/jptcp.2023.30.14.025 |
| 51 | Sköld-Larsson K | 2013 | Sweden | RCT | 64 | 13-18 years | Fluoridated milk | Non-fluoridated milk | De-/remineralisation balance | Positive | Moderate | 10.3109/00016357.2012.696687 |
| 52 | Petersen PE | 2015 | Bulgaria | Cohort | 1498 | 3 years | Fluoridated milk / yogurt | Non-fluoridated milk | Caries development↓ | Positive | Moderate | PMID: 26738215 |
| 53 | Kallagova AR | 2023 | Russia | Observational study / Intervention | 2045 | 3-6 years | Fluoridated milk | Regular milk | ↓Caries increment (deciduous and permanent) ↔ Oral hygiene, salivation rate, oral pH | Positive | Moderate (NIH fair) | 10.51847/0BB2ttxzlA |
| 54 | Chi D | 2016 | Peru | RCT (proof-of-principle) | 153 (ITT) | 6-9 years (schoolchildren) | Xylitol-sweetened milk | Not specified | Caries prevention | Positive | Moderate | PMID: 28327266 |
| 55 | Navit S | 2020 | India | In vivo | 70 (35+35) | 6–14 years | Flavoured milk | None | Salivary pH changes (flavour-dependent) | Neutral | Moderate | 10.5005/jp-journals-10005-1867 |
| 56 | Zare Javid A | 2020 | Iran | RCT | 66 | 18-30 years | *B. lactis* Bb12 yogurt | Placebo | *S. mutans* and *Lactobacillus*↓ | Positive | Moderate | 10.1159/000504164 |
| 57 | Sakaryali Uyar D | 2024 | Turkey | RCT | 58 | 2-12 years | Multi-strain *Bifidobacterium* drops | Not specified | *S. mutans*↓(p=0.01); no *Lactobacillus* effect | Mixed | Moderate | 10.1186/s12903-024-04659-y |
| 58 | Bolla VL | 2023 | India | RCT | 90 | 6-15 years | *B. clausii* UBBC-07 mouth rinse | Placebo | *S. mutans*↓ | Positive | Moderate | 10.4103/aam.aam_95_22 |
| 59 | Lai S | 2021 | Italy | RCT | 68 | 4-14 years (diabetic) | *L. brevis* CD2 lozenge | Placebo | Cariogenic bacteria↓, plaque pH↑, bleeding↓ | Positive | Moderate | 10.1007/s00784-020-03342-0 |
| 60 | Kang MS | 2021 | Korea | RCT | 68 | >20 years | *W. cibaria* CMU tablet | Placebo | Cariview score (high-risk 32.4%→8.8%) | Positive | Moderate | 10.3390/ijerph18094674 |
| 61 | Staszczyk M | 2022 | Poland | RCT | 140 | 3-6 years | *L. salivarius* tablet | Not specified | Caries incidence↓(p<0.001); OHI-S no change | Positive | Moderate | 10.3390/ijerph191912447 |
| 62 | Janiani P | 2022 | India | RCT | 34 | 3-6 years | *L. casei* Shirota (Yakult®) vs. multi-strain powder | Baseline | *S. mutans*↓, plaque score↓(Yakult) | Positive | Moderate | 10.17219/dmp/139731 |
| 63 | Manmontri C | 2020 | Thailand | RCT | 268 | 3.2±0.8 years | *L. paracasei* SD1 milk | Placebo | *S. mutans*↓, *Lactobacillus*↑ | Positive | Moderate | 10.1007/s00784-019-03095-5 |
| 64 | Wattanarat O | 2020 | Thailand | RCT | 268 | 3-6 years | *L. paracasei* SD1 milk (daily or triweekly) | Placebo | *S. mutans*↓, HNP1-3↑, caries progression↓ | Positive | Moderate | 10.1007/s00784-020-03606-9 |
| 65 | Teanpaisan R | 2015 | Thailand | RCT | 122 | 12-14 years | *L. paracasei* SD1 milk powder | Placebo | *S. mutans*↓at 3&6m; caries increment↓in high-risk | Positive | Moderate | 10.3390/dj3020043 |
| 66 | Ghasemi E | 2017 | Iran | RCT | 44 | 6-12 years | *L. rhamnosus* tablet vs. yogurt vs. gum | Baseline | *S. mutans*↓(yogurt and gum) | Positive | Moderate | 10.17796/1053-4628-41.4.257 |
| 67 | Ratna Sudha M | 2020 | India | Double-blind, randomized, placebo-controlled trial | 48 | 5–15 years | Probiotic Bacillus coagulans Unique IS2 (2 billion cfu) chewable tablet, daily for 14 days | Placebo chewable tablet | Statistically significant reduction in salivary and plaque mutans streptococci and lactobacilli counts after 14 days (paired t-test) | Positive | Moderate | 10.1155/2020/8891708 |
| 68 | Rodriguez G | 2016 | Chile | Triple‑blind, placebo‑controlled cluster randomized trial | 205 | 2-3 years | Probiotic milk (*L. rhamnosus* SP1, 10⁷ CFU/mL), 150 mL/day, weekdays for 10 months | Standard milk | ↓ Caries prevalence (54.4% vs 65.8%); ↓ new cavitated lesions (OR=0.35); ↓ mean new cavitated lesions per child (0.58±1.17 vs 1.08±1.70, P<0.01) | Positive | Moderate | 10.1177/0022034515623935 |
| 69 | Taipale T | 2013 | Finland | RCT | 106 | 4 years | *B. animalis* subsp. *lactis* BB-12 lozenge | Placebo | dmft, caries prevalence (not significant) | Neutral | Moderate | 10.1159/000348424 |
| 70 | Duraisamy V | 2021 | India | RCT | 40 | 6-12 years | Multi-strain curd (Nilgiris Dahi Curd) | Control | *S. mutans*↓(p=0.001) | Positive | Moderate | 10.4103/jisppd.jisppd_270_21 |
| 71 | Porksen CJ | 2023 | Denmark | RCT | 288 | 5-9 years | LGG+*L. paracasei*+*L. casei* + arginine lozenge | Placebo | Caries progression (p=0.20, not significant) | Neutral | Moderate | 10.1016/j.jdent.2023.104670 |
| 72 | Ferrer MD | 2020 | Spain | RCT | 59 | 18-65 years | *S. dentisani* CECT 7746 gel | Placebo | Plaque index↓, gingival index↓, salivary flow↑, microbiota shift | Positive | Moderate | 10.3389/fcimb.2020.00465 |
| 73 | Fernando JR | 2019 | Australia | Clinical | 19 | 33 years (mean) | CPP-ACP gum | No gum | Alkali-producing bacteria (S. sanguinis etc.)↑ | Positive | High (COI) | 10.1016/j.jdent.2019.103225 |
| 74 | Martínez-Pabón MC | 2013 | Colombia | Clinical | 130 | 20 years (mean) | Hexitol + CPP-ACP gum | Non-CPP-ACP gum / no gum | Visible plaque↓, calcium/phosphate concentrations↑ | Positive | Moderate | 10.1007/s00784-013-0999-y |
| 75 | Walker G | 2006 | Australia | In situ | 10 | NR (adults) | CPP-ACP milk (2.0-5.0 g/L) | Plain milk | Enamel remineralization (70-148% vs control) | Positive | High (conflict of interest) | 10.1017/S0022029905001482 |
| 76 | Jenkins GN | 1989 | UK | Comparative study (2 experiments) | NR | Adult volunteers | Cheese (+/- biscuit), acidic soft drinks, tea/coffee | Baseline (no cheese / no biscuit / no drink) | Oral fluid Ca²⁺ ↑ (30 → 200-540 μg/mL); oral fluid PO₄³⁻↓; plaque Ca²⁺ ↑ (highly significant); plaque PO₄³⁻ no change | Positive | Moderate (historic) | 10.1159/000261170 |
| 77 | Goncalves FMC | 2025 | Brazil | In situ cross-over | NR | NR (adults) | Fluoride/CPP-ACP/sodium trimetaphosphate toothpaste | Control toothpaste | Early caries remineralization | Positive | Moderate | 10.1016/j.jdent.2025.105618 |
| 78 | Sitthisettapong T | 2015 | Thailand | RCT | 103 | 2.5 – 3.5 years | CPP-ACP toothpaste | Placebo | Enamel caries remineralization (not significant) | Neutral | Moderate | 10.1159/000434728 |
| 79 | Starck E | 2025 | Sweden | RCT | 40 | 6-14 years | Probiotic lozenge | Placebo | No significant difference in oral parameters | Neutral | Moderate | 10.3390/nu17223604 |
