# Supplementary material for: Functional mechanisms, current applications, and future perspectives of dairy products in oral health
Source: Front Public Health. 2026 Aug 31;14:1898007. doi: 10.3389/fpubh.2026.1898007 (PMC13572671; doi:10.3389/fpubh.2026.1898007)
Supplement: Supplementary file 3 [file Table_3.docx]

**Table S3-Risk of bias assessment results for each included study**

| **ID** | **First author** | **year** | **Study design** | **Assessment tool** | **Domain1** | **Domain2** | **Domain3** | **Domain4** | **Domain5** | **Overall judgment** | **Notes** | **Column1** |
| --- | --- | --- | --- | --- | --- | --- | --- | --- | --- | --- | --- | --- |
| 1 | Nase L | 2001 | RCT | RoB 2.0 | Low | Low | Low | Low | Low | Low |  |  |
| 2 | Shen P | 2001 | In situ | Not applicable |  |  |  |  |  | High (COI) | No formal RoB; judged high due to conflict of interest |  |
| 3 | Ahola AJ | 2002 | RCT | RoB 2.0 | Some concerns | Low | Low | Low | Low | Moderate | Randomization unclear |  |
| 4 | Cai F | 2003 | In situ | Not applicable |  |  |  |  |  | High (COI) | Conflict of interest (industry funded) |  |
| 5 | Reynolds EC | 2003 | Clinical | RoB 2.0 (adapted) | Low | Low | Low | Some concerns | High | High | Conflict of interest (industry funded) |  |
| 6 | Iijima Y | 2004 | In situ | Not applicable |  |  |  |  |  | High (COI) |  |  |
| 7 | Itthagarun A | 2005 | In situ | Not applicable |  |  |  |  |  | High (COI) |  |  |
| 8 | Cai F | 2007 | In situ | Not applicable |  |  |  |  |  | High (COI) |  |  |
| 9 | Schirrmeister JF | 2007 | In situ | Not applicable |  |  |  |  |  | Low | Independent study |  |
| 10 | Caglar E | 2008 | RCT | RoB 2.0 | Some concerns | Low | Low | Low | Low | Moderate | Blinding not described |  |
| 11 | Manton DJ | 2008 | In situ | Not applicable |  |  |  |  |  | High (COI) |  |  |
| 12 | Morgan MV | 2008 | In vivo RCT | RoB 2.0 | Low | Low | Low | Low | Some concerns | Moderate | Industry funded but well-conducted |  |
| 13 | Cai F | 2009 | In situ | Not applicable |  |  |  |  |  | High (COI) |  |  |
| 14 | Walker GD | 2009 | In situ | Not applicable |  |  |  |  |  | High (COI) |  |  |
| 15 | Tanaka K | 2012 | Prospective cohort | NOS |  |  |  |  |  |  | Moderate |  |
| 16 | Walker GD | 2010 | In situ | Not applicable |  |  |  |  |  | High (COI) |  |  |
| 17 | Aminabadi NA | 2011 | RCT | RoB 2.0 | Some concerns | Low | Low | Low | Low | Moderate | Randomization unclear |  |
| 18 | Cildir SK | 2012 | RCT crossover | RoB 2.0 | Some concerns | Low | Low | Low | Low | Moderate | Carryover effect not assessed |  |
| 19 | Cochrane NJ | 2012 | In situ | Not applicable |  |  |  |  |  | High (COI) |  |  |
| 20 | Iniesta M | 2012 | RCT | RoB 2.0 | Low | Low | Low | Low | Low | Low |  |  |
| 21 | Campus G | 2014 | RCT | RoB 2.0 | Low | Some concerns | Low | Low | Low | Moderate | Blinding of participants unclear |  |
| 22 | Alp S | 2018 | RCT | RoB 2.0 | Low | Low | Low | Low | Low | Low |  |  |
| 23 | Bhalla M | 2015 | RCT | RoB 2.0 | Some concerns | Some concerns | Low | Low | Low | Low | JADAD score 2 (low quality) |  |
| 24 | Chinnappa A | 2013 | RCT | RoB 2.0 | Some concerns | Low | Low | Low | Low | Moderate |  |  |
| 25 | Llena C | 2015 | RCT | RoB 2.0 | Some concerns | Low | Low | Low | Low | Moderate | Sample size not reported |  |
| 26 | Nozari A | 2015 | RCT | RoB 2.0 | Some concerns | Low | Low | Low | Low | Moderate |  |  |
| 27 | Piwat S | 2020 | RCT | RoB 2.0 | Low | Low | Low | Low | Low | Low |  |  |
| 28 | Srivastava S | 2016 | RCT | RoB 2.0 | Low | Low | Low | Low | Low | Moderate | Double-blind but some concerns, kept moderate |  |
| 29 | Zaki NAA | 2015 | Observational | NIH |  |  |  |  |  |  | Moderate (fair) |  |
| 30 | Hegde A | 2014 | Case-control | NOS |  |  |  |  |  |  | Moderate |  |
| 31 | Neves PAM | 2016 | Observational | NIH |  |  |  |  |  |  | Moderate |  |
| 32 | Nirunsittirat A | 2016 | Prospective cohort | NOS |  |  |  |  |  |  | Moderate |  |
| 33 | Johansson I | 2018 | Prospective cohort | NOS |  |  |  |  |  |  | Moderate |  |
| 34 | Sungkar S | 2020 | Observational | NIH |  |  |  |  |  |  | Moderate |  |
| 35 | Wang J | 2021 | Observational (NHANES) | NIH |  |  |  |  |  |  | Moderate (good) |  |
| 36 | Garcia-Pola M | 2021 | Observational | NIH |  |  |  |  |  |  | Moderate |  |
| 37 | Yardimci H | 2021 | Cross-sectional | NIH |  |  |  |  |  |  | Moderate |  |
| 38 | Olczak-Kowalczyk D | 2021 | Observational | NIH |  |  |  |  |  |  | Moderate |  |
| 39 | Pinto GS | 2014 | RCT | RoB 2.0 | Some concerns | Low | Low | Low | Low | Moderate |  |  |
| 40 | Caglar E | 2014 | RCT | RoB 2.0 | Some concerns | Low | Low | Low | Low | Moderate |  |  |
| 41 | Mahantesha T | 2015 | Pre-post (no control) | NIH pre-post |  |  |  |  |  |  | High | No control group |
| 42 | Ashwin D | 2015 | RCT | RoB 2.0 | Low | Low | Low | Low | Low | Moderate | High loss to follow-up at 6 months, still moderate |  |
| 43 | Lodi CS | 2015 | RCT | RoB 2.0 | Some concerns | Low | Low | Low | Low | Moderate |  |  |
| 44 | Villavicencio J | 2018 | Cluster RCT | RoB 2.0 (cluster) | Low | Low | Low | Low | Low | Moderate | Cluster design but well-conducted |  |
| 45 | Patil RU | 2019 | RCT | RoB 2.0 | Some concerns | Low | Low | Low | Low | Moderate | Tiny sample size (n=10) |  |
| 46 | Sandoval F | 2021 | RCT | RoB 2.0 | Low | Low | Low | Low | Low | Low |  |  |
| 47 | Xu L | 2021 | Pre-post | NIH pre-post |  |  |  |  |  |  | High | No control group (n=6) |
| 48 | Reddy S | 2021 | Interventional | NIH |  |  |  |  |  |  | Moderate |  |
| 49 | Sakhare S | 2021 | RCT | RoB 2.0 | Low | Low | Low | Low | Low | Moderate |  |  |
| 50 | Guru Vishnu C | 2023 | RCT | RoB 2.0 | Some concerns | Low | Low | Low | Low | Moderate | Randomization method not specified |  |
| 51 | Sköld-Larsson K | 2013 | RCT | RoB 2.0 | Low | Low | Low | Low | Low | Moderate |  |  |
| 52 | Petersen PE | 2015 | Cohort | NOS |  |  |  |  |  |  | Moderate |  |
| 53 | Kallagova AR | 2023 | Observational / Intervention | NIH |  |  |  |  |  |  | Moderate (fair) |  |
| 54 | Chi D | 2016 | RCT (proof-of-principle) | RoB 2.0 | Some concerns | Low | Low | Low | Low | Moderate |  |  |
| 55 | Navit S | 2020 | In vivo | NIH |  |  |  |  |  |  | High | No control group |
| 56 | Zare Javid A | 2020 | RCT | RoB 2.0 | Low | Low | Low | Low | Low | Low |  |  |
| 57 | Sakaryali Uyar D | 2024 | RCT | RoB 2.0 | Low | Low | Low | Low | Low | Moderate |  |  |
| 58 | Bolla VL | 2023 | RCT | RoB 2.0 | Low | Low | Low | Low | Low | Moderate |  |  |
| 59 | Lai S | 2021 | RCT | RoB 2.0 | Low | Low | Low | Low | Low | Low |  |  |
| 60 | Kang MS | 2021 | RCT | RoB 2.0 | Low | Low | Low | Low | Low | Low |  |  |
| 61 | Staszczyk M | 2022 | RCT | RoB 2.0 | Low | Low | Low | Low | Low | Low |  |  |
| 62 | Janiani P | 2022 | RCT | RoB 2.0 | Some concerns | Low | Low | Low | Low | Moderate |  |  |
| 63 | Manmontri C | 2020 | RCT | RoB 2.0 | Low | Low | Low | Low | Low | Low |  |  |
| 64 | Wattanarat O | 2020 | RCT | RoB 2.0 | Low | Low | Low | Low | Low | Low |  |  |
| 65 | Teanpaisan R | 2015 | RCT | RoB 2.0 | Low | Low | Low | Low | Low | Low |  |  |
| 66 | Ghasemi E | 2017 | RCT | RoB 2.0 | Low | Low | Low | Low | Low | Moderate |  |  |
| 67 | Ratna Sudha M | 2020 | RCT | RoB 2.0 | Low | Low | Low | Low | Low | Low |  |  |
| 68 | Rodríguez G | 2016 | Cluster RCT | RoB 2.0 (cluster) | Low | Low | Low | Low | Low | Low |  |  |
| 69 | Taipale T | 2013 | RCT | RoB 2.0 | Low | Some concerns | Low | Low | Low | Moderate |  |  |
| 70 | Duraisamy V | 2021 | RCT | RoB 2.0 | Low | Low | Low | Low | Low | Moderate |  |  |
| 71 | Porksen CJ | 2023 | RCT | RoB 2.0 | Low | Low | Low | Low | Low | Low |  |  |
| 72 | Ferrer MD | 2020 | RCT | RoB 2.0 | Low | Low | Low | Low | Low | Low |  |  |
| 73 | Fernando JR | 2019 | Clinical | Not applicable |  |  |  |  |  | High (COI) |  |  |
| 74 | Martínez-Pabón MC | 2013 | Clinical | RoB 2.0 (adapted) | Some concerns | Low | Low | Some concerns | Low | Moderate |  |  |
| 75 | Walker G | 2006 | In situ | Not applicable |  |  |  |  |  | High (COI) |  |  |
| 76 | Jenkins GN | 1989 | Comparative study | Modified |  |  |  |  |  | Moderate | Historic study (pre-CONSORT) |  |
| 77 | Goncalves FMC | 2025 | In situ cross-over | Not applicable |  |  |  |  |  | Moderate |  |  |
| 78 | Sitthisettapong T | 2015 | RCT | RoB 2.0 | Some concerns | Low | Low | Low | Low | Moderate |  |  |
| 79 | Starck E | 2025 | RCT | RoB 2.0 | Low | Low | Low | Low | Low | Low |  |  |
