# Supplementary material for: Functional mechanisms, current applications, and future perspectives of dairy products in oral health
Source: Front Public Health. 2026 Aug 31;14:1898007. doi: 10.3389/fpubh.2026.1898007 (PMC13572671; doi:10.3389/fpubh.2026.1898007)
Supplement: Supplementary file 4 [file Table_4.docx]

**Table S4-PRISMA 2020 checklist**

| **Section and Topic** | **Item #** | **Checklist item** | **Location where item is reported** |
| --- | --- | --- | --- |
| **TITLE** |  |  |  |
| **Title** | 1 | Identify the report as a systematic review. | Title page |
| **ABSTRACT** |  |  |  |
| **Abstract** | 2 | See the PRISMA 2020 for Abstracts checklist. | Abstract (structured) |
| **INTRODUCTION** |  |  |  |
| **Rationale** | 3 | Describe the rationale for the review in the context of existing knowledge. | Introduction (Section 1) |
| **Objectives** | 4 | Provide an explicit statement of the objective(s) or question(s) the review addresses. | Introduction (end of Section 1) |
| **METHODS** |  |  |  |
| **Eligibility criteria** | 5 | Specify the inclusion and exclusion criteria for the review and how studies were grouped for the syntheses. | Methods 2.3 |
| **Information sources** | 6 | Specify all databases, registers, websites, organisations, reference lists, and other sources searched. Specify the date when each source was last searched. | Methods 2.2 |
| **Search strategy** | 7 | Present the full search strategies for all databases, registers and websites. | Supplementary Table S1 |
| **Selection process** | 8 | Specify the methods used to decide whether a study met the inclusion criteria, including how many reviewers screened each record and each report retrieved, whether they worked independently, and details of automation tools. | Methods 2.4 |
| **Data collection process** | 9 | Specify the methods used to collect data from reports, including how many reviewers collected data, whether they worked independently, any processes for obtaining or confirming data from study investigators, and details of automation tools. | Methods 2.4 |
| **Data items** | 10 | List and define all outcomes for which data were sought. Specify whether all results that were compatible with each outcome domain were sought, and if not, the methods used to decide which results to collect. | Methods 2.6; Supplementary Table S2 |
| **Study risk of bias assessment** | 11 | Specify the methods used to assess risk of bias in the included studies, including details of the tool(s) used, how many reviewers assessed each study and whether they worked independently. | Methods 2.5 |
| **Effect measures** | 12 | Specify for each outcome the effect measure(s) (e.g., risk ratio, mean difference) used in the synthesis or presentation of results. | Results 3.7 (narrative synthesis) |
| **Synthesis methods** | 13a | Describe the processes used to decide which studies were eligible for each synthesis (e.g., tabulating study intervention characteristics). | Methods 2.6 |
|  | 13b | Describe any methods required to prepare the data for presentation or synthesis, such as handling of missing summary statistics. | Methods 2.6 |
|  | 13c | Describe any methods used to tabulate or visually display results of individual studies and syntheses. | Results (Table S2, Figure 1) |
|  | 13d | Describe any methods used to synthesize results and provide a rationale for the choice(s). If meta-analysis was performed, describe model(s), heterogeneity assessment, and software. | Methods 2.6 (narrative synthesis) |
|  | 13e | Describe any methods used to explore possible causes of heterogeneity among study results. | Discussion 4.3 |
|  | 13f | Describe any sensitivity analyses conducted to assess robustness of the synthesized results. | Not applicable (no meta-analysis) |
| **Reporting bias assessment** | 14 | Describe any methods used to assess risk of bias due to missing results (reporting biases). | Discussion 4.3 (publication bias) |
| **Certainty assessment** | 15 | Describe any methods used to assess certainty (or confidence) in the body of evidence for an outcome. | Methods 2.5; Discussion 4.3 (GRADE) |
| **RESULTS** |  |  |  |
| **Study selection** | 16 | Describe the results of the search and selection process, from number of records identified to number of studies included, ideally with a flow diagram. | Results 3.1; Figure S1 |
| **Study characteristics** | 17 | Cite each included study and present its characteristics. | Supplementary Table S2 |
| **Risk of bias in studies** | 18 | Present assessments of risk of bias for each included study. | Results 3.6; Supplementary Table S3 |
| **Results of individual studies** | 19 | For all outcomes, present for each study: (a) summary statistics and (b) an effect estimate and its precision, ideally using structured tables or plots. | Supplementary Table S2; Results 3.7 |
| **Results of syntheses** | 20a | For each synthesis, briefly summarise the characteristics and risk of bias among contributing studies and present the results of each synthesis. | Results 3.2-3.5, 3.7 |
|  | 20b | If meta-analysis was not performed, briefly describe the main findings of the comparison(s). | Results 3.7 (quantitative summary) |
| **Reporting biases** | 21 | Present assessments of risk of bias due to missing results (reporting biases) for each synthesis assessed. | Discussion 4.3 |
| **Certainty of evidence** | 22 | Present assessments of certainty (or confidence) in the body of evidence for each outcome. | Discussion 4.3 (GRADE) |
| **DISCUSSION** |  |  |  |
| **Discussion** | 23a | Provide a general interpretation of the results in the context of other evidence. | Discussion 4.1-4.2 |
|  | 23b | Discuss any limitations of the evidence included in the review. | Discussion 4.3 |
|  | 23c | Discuss any limitations of the review processes used. | Discussion 4.3 |
|  | 23d | Discuss implications of the results for practice, policy, and future research. | Discussion 4.4-4.5 |
| **OTHER INFORMATION** |  |  |  |
| **Registration and protocol** | 24a | Provide registration information for the review, including register name and registration number, or state that the review was not registered. | Methods 2.1 (not registered) |
|  | 24b | Indicate where the review protocol can be accessed, or state that a protocol was not prepared. | Methods 2.1 (no protocol prepared) |
| **Support** | 25 | Describe sources of financial or non-financial support for the review, and the role of funders or sponsors. | Funding statement (no funding) |
| **Competing interests** | 26 | Declare any competing interests of review authors. | Conflict of interest statement (none) |
| **Availability of data, code, other materials** | 27 | Report which of the following are publicly available and where they can be found: data collection forms; extracted data; data used for analyses; analytic code; other materials. | Data availability statement |
