# Supplementary material for: Functional mechanisms, current applications, and future perspectives of dairy products in oral health
Source: Front Public Health. 2026 Aug 31;14:1898007. doi: 10.3389/fpubh.2026.1898007 (PMC13572671; doi:10.3389/fpubh.2026.1898007)
Supplement: Supplementary file 5 [file Table_5.docx]

**Table S5-GRADE profile**

| **No. of studies** | **Study design** | **Risk of bias** | **Inconsistency** | **Indirectness** | **Imprecision** | **Other considerations** | **Effect estimate (range)** | **Certainty** |
| --- | --- | --- | --- | --- | --- | --- | --- | --- |
| **38 RCTs** | Randomized trials | Not serious | Serious¹ | Not serious | Not serious | None | 18% to 78% reduction (median 42%) | ●●●◯ MODERATE |
| **22 in situ / RCTs** | Randomized trials / in situ | Very serious² | Serious¹ | Serious³ | Not serious | Publication bias suspected⁴ | 19% to 176% increase in remineralization vs control | ●●◯◯ LOW |
| **1 meta-analysis (19 observational)** | Observational studies | Not serious | Serious⁵ | Not serious | Not serious | None | OR = 0.76 (95% CI 0.66–0.87) | ●●◯◯ LOW |
| ¹ Heterogeneity in probiotic strains, doses, delivery vehicles, and populations. ² Conflict of interest (majority from single research group). ³ Short-term in situ, not clinical caries endpoints. ⁴ >80% from Reynolds’ laboratory. ⁵ Heterogeneity expected; observational design. | | | | | | | | |

GRADE certainty of evidence: ●●●● High, ●●●◯ Moderate, ●●◯◯ Low, ●◯◯◯ Very low. The symbols used in this table (●) are equivalent to ⨁ for compatibility with document rendering.

The certainty ratings reflect confidence in the clinical evidence for each outcome. Mechanistic evidence supporting biological plausibility was considered separately and is discussed in the main text (Section 4.3) and illustrated in Figure 1.
